# Exploration of Mycotoxin Accumulation and Transcriptomes of Different Wheat Cultivars during *Fusarium graminearum* Infection

**DOI:** 10.3390/toxins14070482

**Published:** 2022-07-13

**Authors:** Kailin Li, Dianzhen Yu, Zheng Yan, Na Liu, Yingying Fan, Cheng Wang, Aibo Wu

**Affiliations:** 1 SIBS-UGENT-SJTU Joint Laboratory of Mycotoxin Research, CAS Key Laboratory of Nutrition, Metabolism and Food Safety, Shanghai Institute of Nutrition and Health, University of Chinese Academy of Sciences, Chinese Academy of Sciences, Shanghai 200031, China; likailin2018@sibs.ac.cn (K.L.); dzyu@sibs.ac.cn (D.Y.); zyan@sibs.ac.cn (Z.Y.); liuna@sibs.ac.cn (N.L.); 2 Institute of Quality Standards & Testing Technology for Agro-Products, Xinjiang Academy of Agricultural Sciences, Key Laboratory of Agro-Products Quality and Safety of Xinjiang, Laboratory of Quality and Safety Risk Assessment for Agro-Products (Urumqi), Ministry of Agriculture and Rural Affairs, Urumqi 830091, China; fyyxaas@sina.com (Y.F.); wangchengxj321@sina.com (C.W.)

**Keywords:** wheat, *Fusarium graminearum*, mycotoxin, water activity, temperature, transcriptome

## Abstract

*Fusarium graminearum* is one of the most devastating diseases of wheat worldwide, and can cause Fusarium head blight (FHB). *F. graminearum* infection and mycotoxin production mainly present in wheat and can be influenced by environmental factors and wheat cultivars. The objectives of this study were to examine the effect of wheat cultivars and interacting conditions of temperature and water activity (*a*_w_) on mycotoxin production by two strains of *F. graminearum* and investigate the response mechanisms of different wheat cultivars to *F. graminearum* infection. In this regard, six cultivars of wheat spikes under field conditions and three cultivars of post-harvest wheat grains under three different temperature conditions combined with five water activity (*a*_w_) conditions were used for *F. graminearum* infection in our studies. Liquid chromatography tandem mass spectrometry (LC–MS/MS) analysis showed significant differences in the concentration of Fusarium mycotoxins deoxynivalenol (DON) and its derivative deoxynivalenol-3-glucoside (D3G) resulting from wheat cultivars and environmental factors. Transcriptome profiles of wheat infected with *F. graminearum* revealed the lower expression of disease defense-factor-related genes, such as mitogen-activated protein kinases (MAPK)-encoding genes and hypersensitivity response (HR)-related genes of infected Annong 0711 grains compared with infected Sumai 3 grains. These findings demonstrated the optimal temperature and air humidity resulting in mycotoxin accumulation, which will be beneficial in determining the conditions of the relative level of risk of contamination with FHB and mycotoxins. More importantly, our transcriptome profiling illustrated differences at the molecular level between wheat cultivars with different FHB resistances, which will lay the foundation for further research on mycotoxin biosynthesis of *F. graminearum* and regulatory mechanisms of wheat to *F. graminearum*.

## 1. Introduction

Wheat is an essential food source for humans. Plant diseases and insect pests such as head blight, rusts, powdery mildew, leaf blotch, and wheat curl mite negatively affect the quality and yield of wheat [1,2]. Fusarium head blight (FHB) is a devastating disease that occurs widely in wheat crops in humid and semihumid regions of the world [3]. The average yearly occurrence of FHB has caused severe yield losses [4,5]. During recent decades, many efforts have been deployed to dissect FHB resistance, investigating both the wheat responses to infection and the fungal determinants of pathogenicity [6]. From this, different cultivars of wheat with FHB resistance have been widely conducted as research objects [7,8,9].

FHB can be caused by a variety of *Fusarium graminearum* species complexes (FGSC), and among them, *F. graminearum* is the most prevalent and aggressive pathogen of FHB in wheat [5,10,11]. During infection of wheat, *F. graminearum* can synthesize a large amount of deoxynivalenol (DON) and its derivates, and different *F. graminearum* strains show differences in the capacity of infection and toxin biosynthesis [12,13,14,15]. DON can cause acute physiological effects in humans and animals including vomiting, diarrhea, intestinal inflammation, and gastrointestinal hemorrhage [16]. In some cases, DON can be degraded into masked forms by phase I metabolism or phase II metabolism [17]. Owing to the low toxicity, deoxynivalenol-3-glucoside (D3G) is generally regarded as a detoxification product of DON in plants, and its production is usually related to wheat resistance [18,19,20,21]. Biotransformation of DON in Fusarium-resistant and -susceptible wheat lines shows differences [18,22]. More importantly, it has been reported that D3G can be converted into DON in some food-processing processes, such as dough extrusion, fermentation, and steaming [23,24]. Studies have also shown that some microbiotas in intestines of animals and even humans can rapidly hydrolyze D3G into DON, which provides reasons for much more attention on D3G [25,26,27,28,29,30,31].

In previous studies, the environmental effects on fungal growth and potential mycotoxin contamination were demonstrated by inoculating *F. graminearum*, *F. verticillioides*, *F. langsethiae*, and *F. meridionale* in different cereal matrixes such as maize, oat, and soybean [15,32,33,34]. It has been reported that biosynthesis of DON and D3G is usually affected by environmental temperature, humidity, and hosts [22,34,35,36,37]. However, studies on the effect of *F. graminearum* strains and abiotic factors on mycotoxin production and response mechanisms in wheat-based matrixes are still not comprehensive.

In order to demonstrate that some plant functions and the expression of specific genes are needed to promote FHB, an increasing list of effectors, genes, and mechanisms in the development of FHB have been found using omics approach [20,38]. In particular, the increasing application of transcriptomes has successfully helped researchers map the regulatory responses, which provides an efficient tool for mechanism investigation [39,40,41]. In our study, six cultivars of wheat spikes and three cultivars of post-harvest wheat grains were used for two strains of *F. graminearum* infection to demonstrate differences between wheat cultivars. Further, three different temperature conditions combined with five water activity (*a*_w_) conditions were applied to investigate the interacting effect of wheat cultivars and environmental factors on mycotoxin production. Furthermore, we analyzed the transcriptomic profiles of wheat grains infected with *F. graminearum* F1. Using mycotoxin production analysis combined with transcriptomic analysis, we revealed the differences in toxin concentration and gene expression caused by different *F. graminearum* strains, environmental factors, and wheat cultivars. The results of our study may provide a reference for wheat breeding and wheat storage to reduce the FHB incidence and mycotoxin accumulation in wheat and wheat products, preventing harm to humans. Furthermore, our transcriptome profiling will lay the foundation for further research on mycotoxin biosynthesis and regulatory mechanisms of wheat to *F. graminearum*.

## 2. Results

### 2.1. Evaluation of Toxin Accumulation of Six Wheat Cultivars under Field Conditions

**Figure 1:**
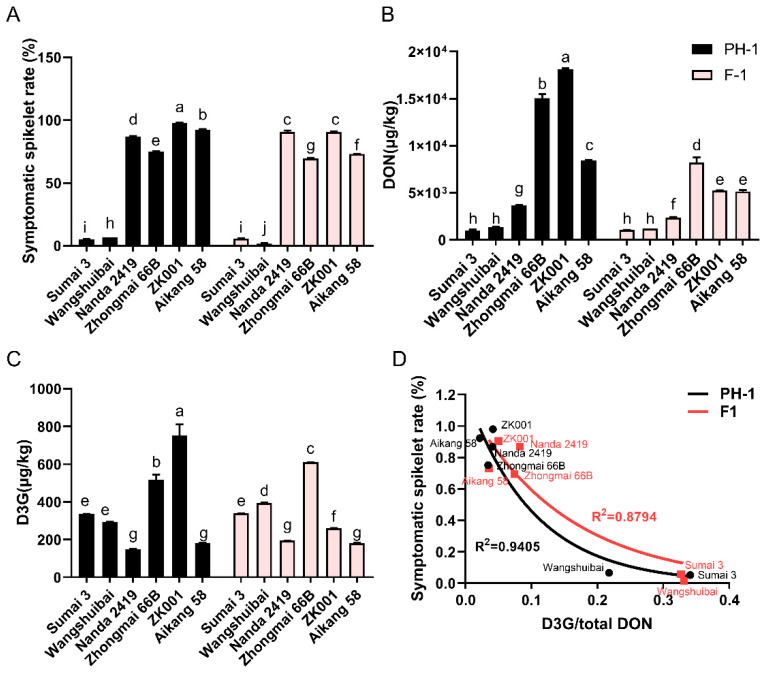
Differences in toxin production levels and symptomatic spikelet numbers between different wheat cultivars. (**A**) Symptomatic spikelet rate of 6 different cultivars of wheat spikes after inoculation with *Fusarium graminearum* PH−1 and *F. graminearum F1*. (**B**) Deoxynivalenol (DON) concentration in 6 varieties of wheat spikes infected with *F. graminearum* PH−1 and *F. graminearum* F1. (**C**) Deoxynivalenol-3-glucoside (D3G) concentration in 6 varieties of wheat spikes infected with *F. graminearum* PH−1 and *F. graminearum* F1. (**D**) Correspondence between symptomatic spikelet rates and D3G/total DON of different wheat cultivars infected with *F. graminearum* PH−1 and F1. Bars with different letters represent significant differences (*p* < 0.05) according to two-way ANOVA; the correspondence was analyzed by Nonlin fit.

Field experiments showed that there were significant differences in resistance to FHB among the wheat cultivars. Both *F. graminearum* PH−1 and *F. graminearum* F1 produced spikelets with blight symptoms on these wheat cultivars (Figure S1). The average symptomatic spikelet numbers of Sumai 3 and Wangshuibai were significantly lower than those of other cultivars of wheat (Figure 1A and Figure S1). In these *F. graminearum* PH−1-infected wheat groups, Sumai 3 was the most resistant, with the lowest symptomatic spikelet rate of 5.21%, while the highest symptomatic spikelet rate was 98.00% of ZK001 (Figure 1A). In the *F. graminearum* F1−infected groups, Wangshuibai was the most resistant cultivar with the lowest symptomatic spikelet rate of 1.54%, while the highest symptomatic spikelet rates were in Nanda 2419 and ZK001, which were up to 90.0% (Figure 1A). Then, wheat spikelets were collected and subjected to mycotoxin determination. The results showed that there were differences in the accumulation of DON between the six varieties of wheat spikelets (Figure 1B). Among them, the concentration of DON produced by *F. graminearum* PH−1 was highest in ZK001 and Aikang 58 followed by Zhongmai 66B (Figure 1B). When inoculated with *F. graminearum* F1, the concentration of DON was highest in Zhongmai 66B followed by ZK001, with values of 8211 µg/kg and 5218 µg/kg, respectively (Figure 1B). The content of DON was lowest in Sumai 3 and Wangshuibai and had no significant difference between these two cultivars when they were infected by *F. graminearum* PH−1 and F1 (Figure 1B). However, compared with DON, the accumulation of D3G was significantly lower (Figure 1C). Furthermore, D3G content was highest in ZK001 and lowest in Nanda 2491 when spikes were infected with *F. graminearum* PH−1 and was highest in Zhongmai 66B and lowest in Aikang 58 and Nanda 2491 when spikes were infected by *F. graminearum* F1 (Figure 1C). By using Nonlinfit analysis, we found that there was a certain negative correlation showing an exponential model change between the ratio of D3G and total DON with symptomatic spikelet rate (Figure 1D). Obviously, the strength of the correlation between D3G/total DON and the symptomatic spikelet rate varies among *F. graminearum* strains (Figure 1D). The R^2^ value was as high as 0.9405 between D3G/total DON and the symptomatic spikelet rate when they were infected by *F. graminearum* PH−1 and 0.8794 between D3G/total DON and the symptomatic spikelet rate when they were infected by *F. graminearum* F1 (Figure 1D). The ratios of D3G/total DON of the FHB-resistant cultivars Sumai 3 and Wangshuibai were higher than other cultivars (Figure 1D).

### 2.2. Accumulation of DON and D3G in F. graminearum PH−1-Infected Wheat Grains under Different a_w_ and Temperature Conditions

**Figure 2:**
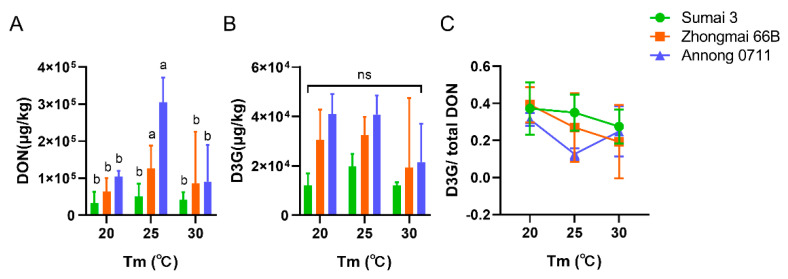
DON (**A**) and D3G (**B**) produced by *F. graminearum* PH−1 and the ratios of D3G/total DON (**C**) of Sumai 3, Zhongmai 66B, and Annong 0711 groups at 20, 25, and 30 °C with water activity (*a*_w_) of 0.99. Bars with different letters represent significant differences (*p* < 0.05) according to two-way ANOVA. The ratio of D3G/total DON was presented as the mean ± SD and analyzed by two-way ANOVA.

To explore the influence of abiotic factors (temperature and *a*_w_) on the accumulations of toxins in wheat grains, we selected Sumai 3 (highly resistant), Annong 0711 (moderately resistant), and Zhongmai 66B (susceptive) for *F. graminearum* PH−1 infection. When the infection time reached one week, DON and D3G had significantly accumulated in the matrixes (Figure 2A,B). Toxin accumulation was most significant at *a*_w_ 0.99, but varied with temperature (Figure 2A). The production trends of DON and D3G were consistent at each temperature, which were higher in susceptive cultivars than resistant cultivars (Figure 2A,B). The concentration of DON reached a maximum at 25 °C (Figure 2A). For D3G, the maximum content was at 25 °C followed by 20 °C (Figure 2B). At 25 °C, the accumulation of DON was approximately 10 times higher in Annong 0711 grains and 5 times higher in Zhongmai 66B grains than in Sumai 3 grains, and the content of D3G was much lower in proportion, reflecting the difference in fungal resistance of wheat cultivars (Figure 2A,B). However, when the *a*_w_ was below 0.99, the accumulation of DON and D3G was not obviously detected in any of the three cultivars, which indicated the importance of *a*_w_ to the accumulation of DONs. Furthermore, the production of DONs at different temperatures did not show significant differences between the three cultivars of wheat grains after infection with *F. graminearum* PH−1 for 7 days (Figure 2A,B). In Figure 2C, the ratio of D3G and total DON did not show a significant difference between these groups.

### 2.3. Accumulation of DON and D3G in F. graminearum F1-Infected Wheat Grains under Different a_w_ and Temperature Conditions

Since wheat contamination in nature is not limited to a single *F. graminearum* strain, we also used *F. graminearum* F1 to infect these cultivars of wheat grains to better illustrate the difference between different FHB-resistance wheat cultivars under different *a*_w_ and temperature conditions. After infection with *F. graminearum* F1 for 7 days, DON and D3G were not obviously detected in Sumai 3 and Zhongmai 66B wheat grains. However, the accumulation of toxins in Annong 0711 was very significant but was lower than that when grains were infected by *F. graminearum* PH−1 (Figure S2).

**Figure 3:**
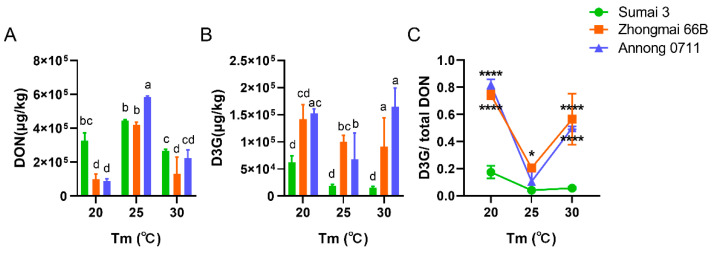
DON (**A**) and D3G (**B**) produced by *F. graminearum* F1 and the ratios of D3G/total DON (**C**) of Sumai 3, Zhongmai 66B, and Annong 0711 groups at 20, 25, and 30 °C with an *a*_w_ of 0.99. Bars with different letters represent significant differences (*p* < 0.05) according to two-way ANOVA. The ratio of D3G/total DON is presented as the mean ± SD and was analyzed by two-way ANOVA. * *p* < 0.05, **** *p* < 0.0001: Zhongmai 66B group and Annong 0711 group, respectively, compared with the Sumai 3 group at the three temperatures.

To clearly measure DON and D3G content differences among the three wheat cultivars, we extended infection time to 14 days. The accumulation of DON and D3G in all three cultivars was much lower under *a*_w_ below 0.99 than above; among these cultivars, DON was highest in Annong 0711 wheat grains but not more than 1500 µg/kg (Table S1). Under the condition of *a*_w_ 0.99, DON in Sumai 3 was highest at 25 °C, followed by 20 °C, while in Zhongmai 66B and Annong 0711, DON was highest at 25 °C, followed by 30 °C (Figure 3A). For D3G, the accumulation in Sumai 3 and Zhongmai 66B reached the highest level at 20 °C, followed by 25 °C. The content of D3G in Annong 0711 was highest at 30 °C, followed by 20 °C (Figure 3B).

The concentration of DON in Zhongmai 66B showed significant differences between 20 °C and 25 °C, 25 °C and 30 °C, and the DON contents in Sumai 3 and Annong 0711 were significantly different under the three temperature conditions (Figure 3A). For D3G, the contents in Sumai 3 showed no differences at the three temperature conditions, but in Zhongmai 66B and Annong 0711 the contents showed a significant difference under the three temperature conditions (Figure 3B).

At 20 °C, the DON and D3G contents showed significant differences between Sumai 3 and Zhongmai 66B, Sumai 3 and Annong 0711, and the D3G content significantly differed in all three cultivars (Figure 3A,B). At 25 °C, the DON content showed a significant difference between Sumai 3 and Annong 0711, Zhongmai 66B and Annong 0711, and the D3G content showed a significant difference between the three cultivars (Figure 3A,B). At 30 °C, the DON content showed a significant difference among the three groups, and the D3G content showed a significant difference between Sumai 3 and Zhongmai 66B, Sumai 3 and Annong 0711(Figure 3A,B). Compared with *F. graminearum* F1-infected Sumai 3, the ratios of D3G and total DON of *F. graminearum* F1-infected Zhongmai 66B and Annong 0711 groups were significantly different at 20 °C and 30 °C (Figure 3C). At 25 °C, only the ratios of Zhongmai 66B and Sumai 3 were statistically different (Figure 3C).

### 2.4. Overview of Differentially Expressed Genes (DEG) between F. graminearum F1-Infected Sumai 3 and Annong 0711 Wheat Grains

**Figure 4:**
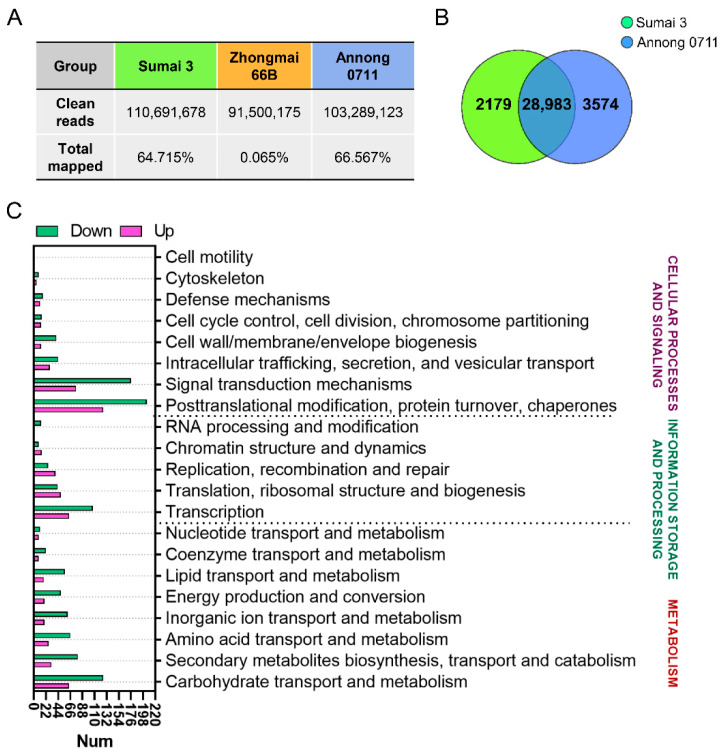
RNA sequencing analysis of Sumai 3 and Annong 0711 wheat gene responses to *F. graminearum* F1. (**A**) Ratio of mapped RNA sequencing reads of the indicated wheat to all sequencing reads after inoculation with *F. graminearum* F1. (**B**) Venn diagram illustration of the genes between the *F. graminearum* F1-treated Sumai 3 group and Annong 0711 group. (**C**) Clusters orthologous groups (COG) annotation of differentially expressed genes (DEGs) between the *F. graminearum* F1-infected Sumai 3 group and Annong 0711 group.

To understand the molecular mechanisms underlying the phenotypic differences between different wheat cultivars, we sequenced the interaction transcriptome of wheat grains and *F. graminearum* F1 under the infection condition of 20 °C and *a*_w_ 0.99 (GEO: GSE188959). Based on the sequencing data, the rates of total mapped clean reads of Sumai 3 and Annong 0711 averaged 64.72% and 66.57%, respectively, which indicated similar infection degrees of the two groups, while the low mapping rate of Zhongmai 66B wheat reads indicated that susceptible varieties were very weak in resistance to *F. graminearum* infection (Figure 4A). Then, Venn analysis was performed for the indicated two groups (Figure 4B). The transcriptome profile of wheat grains showed a significant difference between the infected Sumai 3 and Annong 0711. The results obtained from a differential expression analysis of *F. graminearum* F1-infected Annong 0711 compared with F1-infected Sumai 3 wheat grains showed that 1583 out of 4131 genes were upregulated, while 2548 genes were downregulated, and these genes were annotated based on the clusters orthologous groups (COG) database (Figure 4C).

### 2.5. Gene Ontology (GO) and Kyoto Encyclopedia of Genes and Genomes (KEGG) Enrichment Analyses of DEGs in Wheat

**Figure 5:**
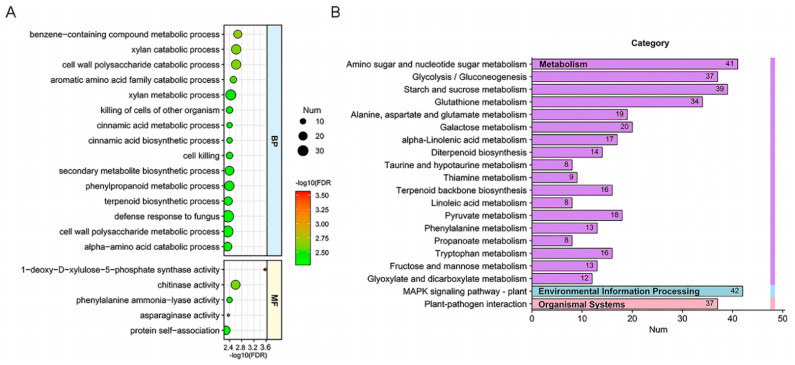
Enrichment analysis of downregulated genes based on gene ontology (GO) and Kyoto encyclopedia of genes and genomes (KEGG) analyses. The top 20 GO categories (**A**) and KEGG pathways (**B**) of the downregulated genes in infected Annong 0711 grains vs. infected Sumai 3 grains were separately grouped and arranged based on their *p* value ranks. The size of the circle represents the gene number.

The GO analysis placed the DEGs into three categories based on their functions: biological process (BP), cellular component (CC), and molecular function (MF). Upregulated DEGs were significantly enriched in eight GO terms; among these terms, functions associated with CC followed by MF accounted for a large proportion (Table S2). KEGG pathway maps included seven categories: metabolism, genetic information processing, environmental information processing, cellular processes, organismal systems, human diseases, and drug development. Upregulated genes were significantly enriched in “glutathione metabolism” and “ribosome biogenesis in eukaryotes” (Table S3). For downregulated genes, the top 20 significantly enriched terms mainly belonged to BP and MF, among which the gene number in “defense response to fungi” was the largest (Figure 5A). According to the KEGG analysis, the top 20 maps of downregulated genes were distributed in metabolism, environmental information processing, and organismal systems categories (Figure 5B).

In these maps, “mitogen-activated protein kinases (MAPK) signaling pathway” and “plant–pathogen interaction” accounted for the largest proportions (Figure 5B). Accordingly, the GO function and KEGG pathway enrichment analyses revealed that compared with the noninfected wheat grains, genes associated with plant defense in the infected Sumai 3 and Annong 0711 grains were both upregulated, while the expression in Annong 0711 wheat grains was significantly lower than that in Sumai 3 (Figure 5 and Table S2). In addition, the expression of genes related to “glutathione metabolism”, “phenylalanine metabolism”, “glycolysis/gluconeogenesis”, “alpha-linolenic acid metabolism”, “galactose metabolism”, “amino sugar and nucleotide sugar metabolism”, and “taurine and hypotaurine metabolism” was also upregulated in the two cultivars of infected wheat grains but was significantly lower in Annong 0711 grains than in Sumai 3 grains, which may illustrate the difference in toxin accumulation between these two wheat cultivars (Figure 5B and Table S3).

**Figure 6:**
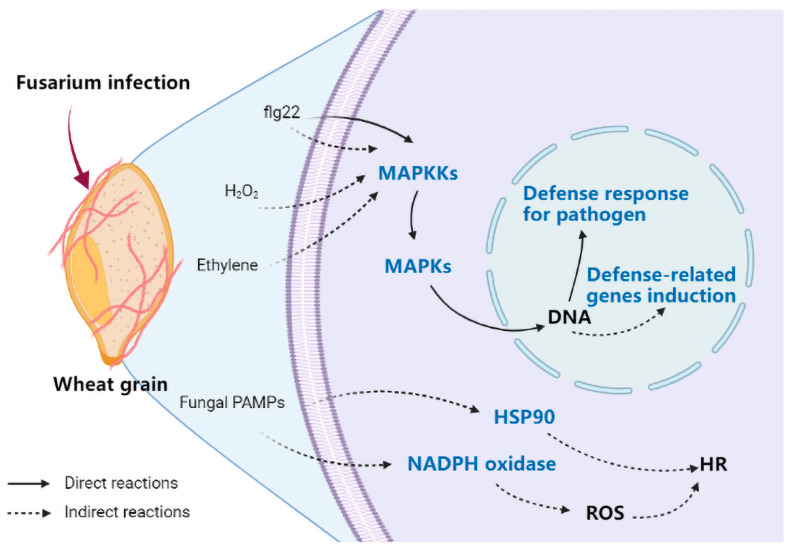
Schematic diagram of Annong 0711 wheat grain genes enriched in the “mitogen activated protein kinases (MAPK) signaling pathway” (map04016) and “plant–pathogen interaction pathway” (map04626). The blue font indicated that the genes that encode the protein were relatively lowly expressed in Annong 0711 compared to Sumai 3 wheat grains.

Investigating the pathways related to plant defense against pathogen infection, we found that the expression of genes related to MAPK and the hypersensitive response (HR) was lower in *F. graminearum* F1-infected Annong 0711 wheat grains than in Sumai 3 wheat grains (Figure 6). The low-level expression of these genes directly or indirectly negatively affected the fungal resistance response, including the defense response for pathogens, the HR, and defense-related downstream gene induction of Annong 0711 wheat.

## 3. Discussion

*Fusarium graminearum* is the major agent of FHB that causes wheat diseases and reduces seed yield worldwide, and different *F. graminearum* strains show differences in infection and toxin production ability [14,42,43,44]. Furthermore, the production of DON and its derivatives was usually affected by environmental conditions such as temperature, humidity and host species [15,22,34,36,37]. In previous studies, the circumstantial effects from temperature and *a*_w_ were demonstrated for potential contamination by inoculating *F. graminearum*, *F. verticillioides*, *F. langsethiae*, and *F. meridionale* in a cereal matrix such as maize, oat, or soybean [15,32,33,34]. However, studies on the effect of *F. graminearum* strains and abiotic factors on mycotoxin production in wheat-based matrixes are still not comprehensive. In our study, we compared the differences in the accumulation of DON and D3G and rates of symptomatic spikelets between six cultivars of wheat spikes that were infected by *F. graminearum* PH−1 and *F. graminearum* F1 under field conditions. We found that there were significant differences in mycotoxin concentration and symptomatic spikelet rates between these wheat cultivars and that the DON and D3G contents were exponentially related to the rates of symptomatic spikelets (Figure 1).

To investigate the effect of abiotic factors on DON and D3G accumulation, we analyzed the mycotoxin assay using three wheat cultivars of harvested wheat grains with known resistance to FHB under indicated temperature and *a*_w_ conditions in a laboratory. Then, we found that the concentration of DON and D3G was significantly increased in Sumai 3, Zhongmai 66B, and Annong 0711 wheat grains that were infected by *F. graminearum* PH−1 for 7 days, while the compounds did not show a significant difference under different temperatures between these cultivars due to the high pathogenicity of PH−1 (Figure 2A,B). Additionally, when the wheat grains were infected by *F. graminearum* F1 for 7 days, the accumulation of DON and D3G only occurred in Annong 0711 wheat (Figure S2). Accordingly, the accumulation of DON and D3G strongly related to the infectivity, genotype and chemotype of *F. graminearum* strains. When the *F. graminearum* F1 infection time was extended to 14 days, the accumulation of DON and D3G and the ratio of D3G to DON showed significant differences between these wheat cultivars but had no relation with the FHB resistance of cultivars (Figure 2C,D and Figure 3 and Table S1). We speculated that under optimal inoculation conditions, the toxin concentration was more affected by the nutrient content in wheat grains.

In order to explore the changes at the molecular level during interaction, we employed RNA-Seq to perform a transcriptomic study and analyzed the changes in gene expression in *F. graminearum* F1-Sumai 3, *F. graminearum* F1-Zhongmai 66B, and *F. graminearum* F1-Annong 0711 wheat grains. Our results had demonstrated significantly DEGs between the *F. graminearum* F1-Sumai 3 and *F. graminearum* F1-Annong 0711 libraries. In previous studies, MAPK genes have been investigated in the plant response to fungal pathogens [45]. It has been reported that DON exerts its effects at the cellular level by activating MAPK through a process known as the ribotoxic stress response, and the outcome of DON-associated MAPK activation is dose- and duration-dependent [16]. In the transcriptome assay, we found that 42 MAPK genes were upregulated in the infected wheat grains; however, compared with infected Sumai 3, the expression of these genes was lower in infected Annong 0711 grains, which is consistent with the DON concentration result (Figure 3 and Figure 5 and Table S3). The hypersensitivity response (HR) is found in all higher plants and is an extremely effective component of the plant immune system [46]. In our study, the expression of genes related to the defense response, especially HR, such as heat shock protein 90 (HSP90) and NADPH oxidase, was also lower in Annong 0711 wheat grains (Figure 5A and Figure 6). These results indicated that the difference in FHB resistance between Sumai 3 and Annong 0711 wheat cultivars was associated with the wheat HR to *F. graminearum*. However, the numbers of DEGs between *F. graminearum* F1 on Sumai 3 and Annong 0711 and enriched pathways were very small (Table S4).

In conclusion, our results illustrated the effect of wheat cultivars, temperature and water activity on mycotoxin production by combining field and laboratory treatments, which will be beneficial in determining the conditions of the relative level of risk of contamination with mycotoxins and providing control strategies to reduce the risk of the occurrence of mycotoxins in pre- and post-harvest wheat. Furthermore, our transcriptome results demonstrated molecular changes in wheat with different FHB resistance and *F. graminearum*, which will lay the foundation for further research on mycotoxin biosynthesis of *F. graminearum* and the regulatory mechanisms of wheat to *F. graminearum*.

## 4. Materials and Methods

### 4.1. Wheat Sample and F. graminearum Strains

Wheat cultivars Sumai 3, Wangshuibai, Zhongmai 66B, ZK001, Aikang 58, and Nanda 2419 were provided by the Hefei Institute of Physical Science, Chinese Academy of Sciences (Table 1). *F. graminearum* F1 and *F. graminearum* PH−1 species were donated by Huazhong Agricultural University. All *F. graminearum* strains were stored as spore suspensions in 20% glycerol at −80 °C.

### 4.2. Chemicals and Reagents

DON and D3G standard solution were purchased from Sigma-Aldrich (St. Louis, MO, USA). Ultrapure water (18.2 MΩ cm) used in our experiments was supplied by Millipore (Bedford, MA, USA). Acetonitrile and methanol (HPLC-grade) were purchased from Honeywell (Shanghai, China). Formic acid (HPLC-grade) was obtained from Anpel (Shanghai, China). Potato dextrose agar medium (PDA) was purchased from BD Difco (San Diego, CA, USA).

### 4.3. Inoculation and Incubation Conditions

Florets of 6 cultivars of wheat including Sumai 3, Wangshuibai, Zhongmai 66B, ZK001, Aikang 58, and Nanda 2419 were used for *F. graminearum* PH−1 and *F. graminearum* F1 infection. Then, 20 μL of *F. graminearum* spore suspension (5 × 10^5^ per mL) was inoculated on the florets (Table 1). Symptomatic spikelets on wheat spikes were measured at 21 days post inoculation, and the rate of symptomatic spikelets was calculated based on the following formula:

Symptomatic spikelets rate (%) = (number of symptomatic spikelets/total wheat spikes number) × 100(1)

For laboratory conditions, one portion of every 25 g of post-harvest grains was irradiated at 8 kGy using a cobalt radiation source and then stored aseptically at 4 °C before utilization. One portion for every 25 g of Sumai 3, Zhongmai 66B, and Annong 0711 wheat grain was plated into a 100 mL sterile conical flask, and the initial *a*_w_ of the wheat grain was 0.572, which was confirmed by using a Novasina Labmaster-Neo water activity meter (Novasina, Inc., WA, Swit). Then, sterile distilled water was added to rehydrate to the required *a*_w_ (*a*_w_: 0.80, 0.85, 0.9, 0.95, and 0.99). Flasks were subsequently refrigerated at 4 °C for 72 h with periodic shaking to ensure uniform absorption and equilibration of water. After three days of equilibration, the wheat grains were inoculated centrally with 4 mm agar plugs taken from the margin of 7-day-old colonies of *F. graminearum* grown on PDA at 25 °C. For all temperatures (20, 25, 30 °C) and *a*_w_ treatments, three replicates per strain were used. The total number of treatments was 3 wheat cultivars × 2 *F. graminearum* strains × 3 temperatures conditions × 5 *a*_w_ conditions × 3 replicates (Table 1). All treatment groups were dried at 60 °C after 7 or 14 days post inoculation and stored at −20 °C until mycotoxin extraction was carried out.

### 4.4. Mycotoxin Extraction

Mycotoxin extractions were determined according to a published method with minor modifications [47]. All samples were ground into a homogenized powder, weighed into 50 mL centrifuge tubes, and then mixed with 10 mL of acetonitrile/water (84:16, *v*/*v*). The tubes were shaken at 2500 rpm/min at 25 °C in an orbital shaker for 60 min and then ultrasonicated for 40 min. Then, tubes were centrifuged at 4000 rpm/min for 30 min. Then, 2 mL of supernatant was transferred to a 15 mL centrifuge tube, 150 mg of anhydrous magnesium sulphate was added, and the tube was vortexed. Then, the supernatant was transferred to a new 15 mL centrifuge tube and 1 mL of n-hexane was added to a biosafety cabinet for degreasing, shaken vigorously, centrifuged to remove n-hexane, and completely dried in a stream of nitrogen. All dried extracts were dissolved in 1 mL of acetonitrile:water (20:80 *v*/*v*). The purified supernatant was filtered through a 0.22 μm nylon filter and stored in sampler vials at −20 °C until LC–MS/MS analysis.

### 4.5. Mycotoxin Determination by LC–MS/MS

LC–MS/MS analysis was performed as described by Yu et al. [15]. Mycotoxins were quantified by an Accela 1250 UPLC system (Thermo Fisher Scientific, San Jose, CA, USA) coupled to a TSQ Vantage^TM^ (Thermo Fisher Scientific, San Jose, CA, USA) triple-stage quadruple mass spectrometer. An Agilent Extend C18 chromatographic column (100 mm × 4.6 mm, 3.5 μm) was used at a flow rate of 0.35 mL/min at 30 °C and with a 10 μL injection volume. The mobile phase consisted of 5 mM ammonium acetate (A) and 100% methanol (B). The gradient was as follows: 0 min 15% B, 1 min 15% B, 6.5 min 90% B, 8.5 min 90% B, 9 min 15% B, and 12 min 15% B. Mass spectrometry analysis was carried out in both positive (ESI + 3.5 kV) and negative (ESI − 2.5 kV) ionization modes using selected reaction monitoring (SRM). For the MS/MS analysis, both the vaporizer and capillary temperatures were 300 °C, the sheath gas pressure was 50 psi, and the aux gas pressure was 5 psi. Raw data were analyzed using Xcalibur™ software (Thermo Fisher Scientific, San Jose, CA, USA, 2011).

The ratio of D3G and total DON was calculated based on the following formula:

D3G/total DON = mD3G/(mDON + nD3G ∗ MDON)(2)

### 4.6. Total RNA Extraction

The total RNA of the mixture samples of Sumai 3, Zhongmai 66B, and Annong 0711 wheat and *F. graminearum* F1 for 14 days was extracted using Plant RNA Purification Reagent for plant tissue (Invitrogen, Carlsbad, CA, USA) according the manufacturer’s instructions, respectively. Genomic DNA was removed by DNaseI (Takara, Beijing, China). The RNA quality and concentration were determined using a NanoDrop 2000 (Agilent Technologies, Santa Clara, CA, USA).

### 4.7. Library Preparation and Sequencing

The RNA-seq transcriptome library was prepared using a TruSeq^TM^ RNA sample preparation kit from Illumina (San Diego, CA, USA) and 1 μg of total RNA. Second, double-stranded cDNA was synthesized using a SuperScript double-stranded cDNA synthesis kit (Invitrogen, CA, USA), 300 bp fragmented mRNA, and random hexamer primers. The synthesized cDNA was then subjected to end repair and adaptor ligation according to Illumina’s library construction protocol. Then, cDNA target fragments of 300 bp were amplified using Phusion DNA polymerase (NEB) for 15 PCR cycles. After quantification by TBS380, the paired-end sequencing library was sequenced using the Illumina HiSeq Xten sequencer (2 × 150 bp read length) [48].

### 4.8. Read Mapping and DEG Analysis

The raw paired-end reads were optimized by SeqPrep (https://github.com/jstjohn/SeqPrep (accessed on 7 June 2022)) and Sickle (https://github.com/najoshi/sickle, accessed on 7 June 2022) using default parameters to obtain clean reads. Then, clean reads were separately aligned to the reference genome with orientation mode using HISAT2 (http://ccb.jhu.edu/software/hisat2/index.shtml, accessed on 7 June 2022) software [49]. Gene abundances were quantified using RSEM (http://deweylab.biostat.wisc.edu/rsem/, accessed on 7 June 2022) [50]. A DEG analysis was performed using DESeq2 with a |log2FC| > 1 and BH-corrected *p* value ≤ 0.05 [51].

### 4.9. Functional Annotation and Enrichment

A Clusters of Orthologous Groups of proteins (COG) annotation analysis was performed using HMMER [52]. Gene Ontology (GO) functional enrichment and Kyoto Encyclopedia of Genes and Genome (KEGG) pathway analysis were carried out by Goatools (https://github.com/tanghaibao/Goatools, accessed on 7 June 2022) and KOBAS (http://kobas.cbi.pku.edu.cn/home.do, accessed on 7 June 2022) at a *p* value ≤ 0.05 or corrected *p* value ≤ 0.05 [53,54].

### 4.10. Quantitative Real-Time PCR Analysis

Total RNA of the mixed culture samples of Sumai 3, Zhongmai 66B, and Annong 0711 wheat and *F. graminearum* F1 for 14 days were prepared using TRIzol reagent (Invitrogen, CA, USA). cDNA was synthesized using PrimeScript™ RT reagent Kit with gDNA Eraser (Takara, Beijing, China). Each sample was quantified using TB Green Premix Ex Taq II (Takara, Beijing, China) following the instructions of the manufacturer and applied to the QuantStudio™ Real-Time PCR System (Applied Biosystems™). The primers employed in this experiment were listed in Table S5. Actin was used as an internal control in this experiment. The 2^−ΔΔCt^ method was used to calculate the expression level and three replicates were employed for every gene [55].

### 4.11. Statistical Analysis

The mycotoxin concentration analysis was constructed using GraphPad Prism 8 (GraphPad Software Inc., San Diego, CA, USA) by two-way ANOVA. Post hoc Tukey’s testing was used to evaluate changes between groups. A *p* value < 0.05 was considered statistically significant.

## Figures and Tables

**Table 1:** List of the *F. graminearum* strains, wheat cultivars, and experimental conditions used for infection.

Strain	Wheat Cultivar	FHB Resistance	Conditions	Subject
*F. graminearum* PH−1 *F. graminearum* F1	Sumai 3	Resistant	Field conditions	Wheat spikes * [9]
	Wangshuibai	Resistant		
	ZK001	Moderately Resistant		
	Nanda 2419	Moderately Resistant		
	Aikang 58	Susceptible		
	Zhongmai 66B	Susceptible		
*F. graminearum* PH−1 *F. graminearum* F1	Sumai 3	Resistant	*a*_w_: 0.80, 0.85, 0.9, 0.95, 0.99 T (°C): 20, 25, 30 [15]	Post-harvest wheat grains
	Annong 0711	Moderately Resistant		
	Zhongmai 66B	Susceptible		

* *F. graminearum* spores were inoculated on wheat florets.

## Data Availability

The data that support the findings of this study are available from the corresponding author upon reasonable request.

## Supplementary Materials

The following supporting information can be downloaded at: https://www.mdpi.com/article/10.3390/toxins14070482/s1, Figure S1: Representative images of 6 different cultivars of wheat spikelets at 21 days after inoculation with *F. graminearum* PH−1 and *F. graminearum* F1; Figure S2: The accumulation of DON and D3G in Annong 0711 grains that were infected by *F. graminearum* F1 at different temperatures and *a*_w_ for 7 days; Table S1: DON and D3G accumulation in Sumai 3, Zhongmai 66B, and Annong 0711 wheat grains infected by *F. graminearum* F1 under different conditions; Table S2: GO and KEGG enrichment analysis of upregulated genes of infected Annong 0711 wheat grains; Table S3: Significantly enriched (corrected *p* value < 0.05) KEGG pathways of upregulated genes in infected wheat grains; Table S4: GO and KEGG enrichment analysis of DEGs of *F. graminearum* F1 in Sumai 3 and Annong 0711 wheat grains; Table S5: Primer sequences used for RT-qPCR amplification of the differentially expressed genes selected for validation.

## Abbreviations

FHB	Fusarium head blight
DON	deoxynivalenol
D3G	deoxynivalenol-3-glucoside
HPLC	high performance liquid chromatography
LC-MS/MS	liquid chromatography tandem mass spectrometry
*a*_w_	water activity
DEG	differentially expressed gene
COG	Clusters Orthologous Groups
GO	Gene Ontology
KEGG	Kyoto Encyclopedia of Genes and Genome
BP	biological process
CC	cellular component
MF	molecular function
MAPK	mitogen activated protein kinases
HR	hypersensitivity response
