# Supplementary material for: Exploration of Mycotoxin Accumulation and Transcriptomes of Different Wheat Cultivars during Fusarium graminearum Infection
Source: Toxins (Basel). 2022 Jul 13;14(7):482. doi: 10.3390/toxins14070482 (PMC9318452; doi:10.3390/toxins14070482)
Supplement: Supplementary file 1 [file toxins-14-00482-s001.zip › toxins-1785152-supplementary.pdf]

Supplementary Materials: Exploration of Mycotoxin Accumulation and Transcriptomes of Different Wheat Cultivars during *Fusarium graminearum* Infection

Kailin Li, Dianzhen Yu, Zheng Yan, Na Liu, Yingying Fan, Cheng Wang and Aibo Wu

Table S1. DON and D3G accumulation in Sumai 3, Zhongmai 66B, and Annong 0711 wheat grains infected by *F. graminearum* F1 under different conditions.

Sumai 3							
	DON (μg/kg)				D3G (μg/kg)		
	20 ℃	25 ℃	30 ℃		20 ℃	25 ℃	30 ℃
0.80	-	-	-	0.80	-	-	-
0.85	-	-	-	0.85	-	-	-
0.90	272.51	242.74	263.61	0.90	32.28	30.29	30.09
0.95	53.79	100.07	54.58	0.95	31.83	32.78	31.53
**Zhongmai 66B**							
	DON (μg/kg)				D3G (μg/kg)		
	20 ℃	25 ℃	30 ℃		20 ℃	25 ℃	30 ℃
0.80	-	-	-	0.80	-	-	-
0.85	-	-	-	0.85	-	-	-
0.90	-	177.80	183.43	0.90	34.53	30.43	31.44
0.95	115.68	76.58	935.57	0.95	33.58	-	-
**Annong 0711**							
	DON (μg/kg)				D3G (μg/kg)		
	20 ℃	25 ℃	30 ℃		20 ℃	25 ℃	30 ℃
0.80	-	-	-	0.80	-	-	-
0.85	-	-	-	0.85	-	-	-
0.90	1,285.98	1,083.18	969.50	0.90	79.86	71.80	69.83
0.95	1,014.35	894.39	814.68	0.95	83.74	90.55	76.09

- Mean levels of toxin in wheat grains were too small to quantify.

Table S2. GO and KEGG enrichment analysis of upregulated genes of infected Annong 0711 wheat grains.

Category	GO/KEGG ID	Description	Number	Corrected P value
MF	GO:0030145	manganese ion binding	25	0.005
	GO:0045735	nutrient reservoir activity	24	0.011
	GO:0042973	glucan endo-1,3-beta-D-glucosidase activity	9	0.007
	GO:0016623	oxidoreductase activity, acting on the aldehyde or oxo group of donors, oxygen as acceptor	6	0.034
CC	GO:0005618	cell wall	40	0.016
	GO:0030312	external encapsulating structure	40	0.016
BP	GO:0006076	(1->3)-beta-D-glucan catabolic process	6	0.007
	GO:0045041	protein import into mitochondrial intermembrane space	6	0.028
Metabolism	map00480	Glutathione metabolism	20	0.024
Genetic Information Processing	map03008	Ribosome biogenesis in eukaryotes	15	0.032

Table S3. Significantly enriched (corrected P value < 0.05) KEGG pathways of upregulated genes in infected wheat grains.

**Category**	**ID**	Description	Gene number	
			Sumai 3	Annong 0711
Metabolism	map00480	Glutathione metabolism	171	169
	map00400	Phenylalanine, tyrosine and tryptophan biosynthesis	59	55
	map00360	Phenylalanine metabolism	72	70
	map00010	Glycolysis / Gluconeogenesis	132	128
	map00020	Citrate cycle (TCA cycle)	59	61
	map00592	alpha-Linolenic acid metabolism	63	64
	map00350	Tyrosine metabolism	62	67
	map00600	Sphingolipid metabolism	45	43
	map00950	Isoquinoline alkaloid biosynthesis	43	47
	map00052	Galactose metabolism	67	68
	map00520	Amino sugar and nucleotide sugar metabolism	120	125
	map00430	Taurine and hypotaurine metabolism	21	21
	map00965	Betalain biosynthesis	25	25
	map00130	Ubiquinone and other terpenoid-quinone biosynthesis	56	57
	map00620	Pyruvate metabolism	70	no
	map00564	Glycerophospholipid metabolism	91	103
	map00250	Alanine, aspartate and glutamate metabolism	53	no
	map00220	Arginine biosynthesis	36	38
	map00760	Nicotinate and nicotinamide metabolism	21	23
	map00901	Indole alkaloid biosynthesis	19	19
	map00908	Zeatin biosynthesis	40	44
	map00920	Sulfur metabolism	32	no
	map00380	Tryptophan metabolism	69	no
	map00450	Selenocompound metabolism	22	25
	map00270	Cysteine and methionine metabolism	83	no
Organismal Systems	map04626	Plant-pathogen interaction	200	209
Environmental Information Processing	map04016	MAPK signaling pathway - plant	182	188
	map02010	ABC transporters	44	47
	map03015	mRNA surveillance pathway	no	96

Note: “no” represented not significantly enriched (corrected P value > 0.5).

Table S4. GO and KEGG enrichment analysis of DEGs of F. *graminearum* F1 in Sumai 3 and Annong 0711 wheat grains.

Change	Category	GO/KEGG ID	Description	Number	Corrected P value
Up	CC	GO:0005576	extracellular region	4	0.04
	BP	GO:0009405	pathogenesis	3	0.01
		GO:0044419	interspecies interaction between organisms	3	0.04
	Metabolism	map00220	Arginine biosynthesis	2	0.01
		map00400	Phenylalanine	2	0.01
		map00250	Alanine, aspartate and glutamate metabolism	2	0.02
		map00360	Phenylalanine metabolism	2	0.03
		map00998	Biosynthesis of various secondary metabolites - part 2	1	0.003
		map00330	Arginine and proline metabolism	2	0.004
		map00270	Cysteine and methionine metabolism	2	0.004
		map00350	Tyrosine metabolism	2	0.049
Down	Metabolism	map00100	Steroid biosynthesis	3	0.006

Table S5. Primers sequences used for RT-qPCR amplification of the differentially expressed genes selected for validation.

Gene ID	Primers sequences
actin	F:5′- CACTGGAATGGTCAAGGCTG -3′<br>AGTTGGTCGGGTCTCTTCTAAATG<br>R:5′- CTCCATGTCATCCCAGTTG-3′
TraesCS5A02G500800	F:5'- ATGGAGGGCAGGGAGGAGAA -3'<br>R:5'- CGCGTCATCGGCTTCATCTC -3'
TraesCS5B02G336300	F:5'- CACGAGGAGGTTGTGCTGATGC -3'<br>R:5'- GGCGATGAGGAAGTTGCTGGTG -3'
TraesCS4A02G454700	F:5'- GCCGCAGGAAAGAGCCGATAAG -3'<br>R:5'- GAGAGAGCCACCGCCAAAGAAC -3'
TraesCS2A02G546600	F:5'- TGAAGAGGCTGACATTGTTCGGTTG -3'<br>R:5'- ACAAGAGTGGAGCAGGAGGATGG -3'
TraesCS7B02G017200	F:5'- GCTCATCTTCTGCACGGAGTCTATG -3'<br>R:5'- AAGCAATCGGGTCCTTGACATCTTC -3'
TraesCS2D02G518200	F:5'- CCACGCCGATGATAGGTTTGAGG -3'<br>R:5'- AGCCTTGCCAGTCTTGTTGAGATTC -3'
TraesCS3A02G024900	F:5'- CTAGCGATGAGAGCGACAGTGATG -3'<br>R:5'- GACCTTGAAGAGTGACGGCGATG -3'
TraesCS3D02G412300	F:5'- GGTGGACTACTGCTCGGAGGAG -3'<br>R:5'- TCTCCCTGACGATGTGCTCCTG -3'
TraesCS5A02G090600	F:5'- GGTGATCAAGACGGAGCTGCTG -3'<br>R:5'- GCCTCTCGTACTCCACCCTGAG -3'
TraesCS7A02G090100	F:5'- CGGGGCAGGAGATGTCCTACAC -3'<br>R:5'- CGTAGCAGCAGAAGCAGCACAG -3'
TraesCSU02G146600	F:5'- ATCTCTGTCATCGACGGCTTCAAC -3'<br>R:5'- CAGGCGTGGTTCTTGGTGTCATC -3'

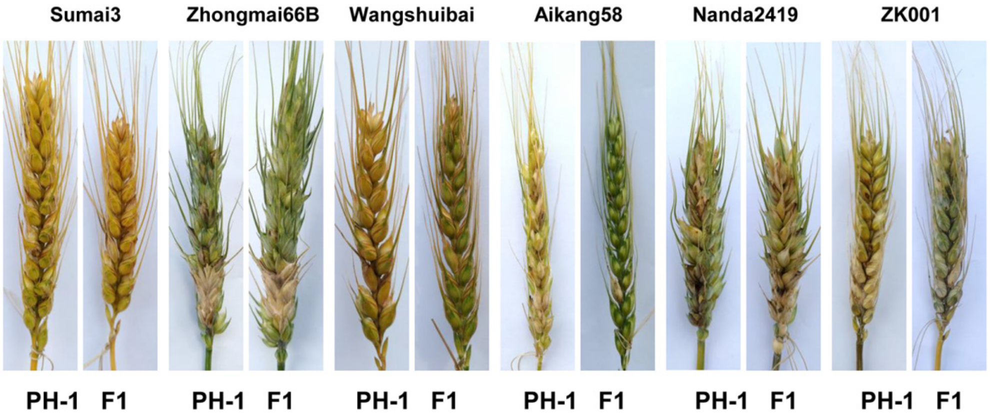

Figure S1. Representative images of 6 different cultivars of wheat spikelets at 21 days after inoculation with *F. graminearum* PH−1 and *F. graminearum* F1.

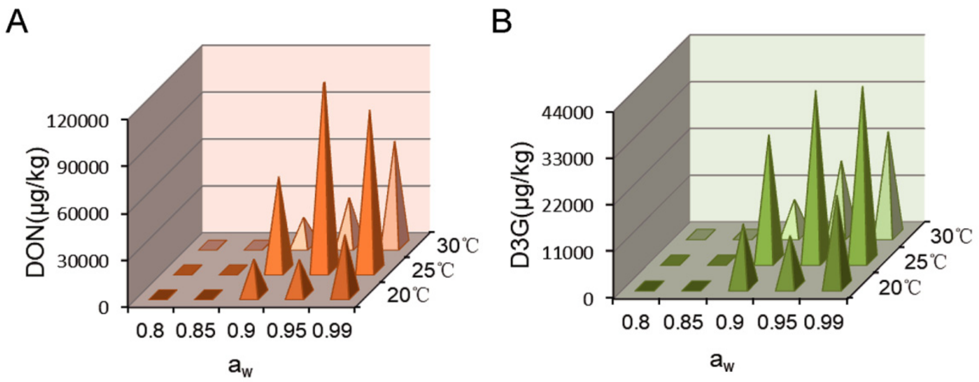

Figure S2. The accumulation of DON and D3G in Annong 0711 grains that were infected by *F. graminearum* F1 at different temperatures and a_w for 7 days.
